# Effect of no eyeglasses sales on the quality of eye care: an experimental evidence from China

**DOI:** 10.1186/s12889-024-17882-7

**Published:** 2024-02-09

**Authors:** Nan Wang, Yangyuan Li, Shichong Wu, Yunjie Liu, Jingchun Nie, Junhao Wu, Zulihumaer Reheman, Jinbiao Ye, Jie Yang

**Affiliations:** 1 Center for Experimental Economics in Education, Shaanxi Normal University, Xi’an, China; 2 Department of Statistics, School of Economics, Xiamen University, Xiamen, China

**Keywords:** Accuracy, Standardized patient, Quality, Eye care, China, Refraction, Separating drug sales from hospital revenue

## Abstract

**Background:**

Eye examinations and eyeglasses acquisition are typically integrated into a cohesive procedure in China. We conducted a randomized controlled trial using incognito standardized patient (SP) approach to evaluate the impact of separating eyeglasses sales on the accuracy of final prescription.

**Methods:**

52 SPs were trained to provide standardized responses during eye examinations, and undergoing refraction by a senior ophthalmologist at a national-level clinical center. SPs subsequently received eye examinations at 226 private optical shops and public hospitals in Shaanxi, northwestern China. The visits were randomly assigned to either control group, where SPs would typically purchase eyeglasses after refraction, or treatment group, where SPs made an advance declaration not to purchase eyeglasses prior to refraction. The dioptric difference between the final prescriptions provided by local refractionists and expert in the better-seeing eye was determined using the Vector Diopteric Distance method, and the completeness of exams was assessed against national standards. Multiple regressions were conducted to estimate the impact of no eyeglasses sales on the accuracy of the final prescription of local refractionists, as well as the completeness of examinations.

**Results:**

Among 226 eye exams (73 in public hospitals, 153 in private optical shops), 133 (58.8%) were randomized to control group and 93 (41.2%) to no eyeglasses sales group. The inaccuracy rate of final prescriptions provided by local refractionists (≥ 1.0 D, experts’ final prescription as the reference) was 25.6% in control group, while 36.6% in no-sale group (*P* = 0.077). The likelihood of providing inaccurate final prescriptions was significantly higher in no-sale group compared to control group (OR = 1.607; 95% CI: 1.030 to 2.508; *P* = 0.037). This was particularly evident in private optical shops (OR = 2.433; 95% CI: 1.386 to 4.309; *P* = 0.002). In terms of process quality, the no-sale group performed significantly less subjective refraction (OR = 0.488; 95% CI: 0.253 to 0.940; *P* = 0.032) and less testing SP’s own eyeglasses (OR = 0.424; 95% CI: 0.201 to 0.897; *P* = 0.025). The duration of eye exams was 3.917 min shorter (95% CI: -6.798 to -1.036; *P* = 0.008) in no-sale group.

**Conclusions:**

Separating eyeglasses sales from optical care could lead to worse quality of eye care. Policy makers should carefully consider the role of economic incentives in healthcare reform.

## Background

The rising expenditures of healthcare systems worldwide, including in China, are a major challenge due to factors such as population aging and advancements in medical technology [1]. However, evidence suggests that heavy reliance on profits from markups on drugs and medical consumables has also contributed to the steep growth [2]. In 2019, China’s total health expenditures accounted for 6.58% of GDP, up from 5.03% in 2009 [3, 4], with drug sales alone accounting for 34.1% of total medical revenues in 2019 [5]. This distorted health financing mechanism has incentivized over-prescription and excess use of consumables, highlighting the issue of information asymmetry [6].

Efforts to control healthcare costs have led to exploring the separation of drug sales from hospital revenue as a significant avenue for healthcare reform. This policy aims to limit drug overuse and reduce inappropriate incentives within the medical field [5]. Studies have shown that this reform has the potential to significantly change hospital revenue structure and reduce patients’ medical expenses [5, 7–9]. However, the impact of separating drug sales from hospital revenue on healthcare quality remains unexplored, partly due to the difficulties in measuring the quality of healthcare [10–12]. It is important to investigate whether reduced incentives resulting from this separation could potentially affect overall healthcare quality, as doctors’ performance may be affected by changes in incentives.

Myopia is a common vision problem in China and has become a major public health concern. The number of myopia cases has significantly increased over the past few decades, affecting over 600 million individuals in China [13, 14]. Eyeglasses are a cost-effective and efficient solution for improving visual function. Eye examinations and the purchase of eyeglasses are typically integrated into a unified procedure, whether individuals seek eye care in public hospitals or private optical shops. Eyeglasses sales generate the most profit for optical shops and hospitals, as well as hospitals relying on revenue (profit) from drug sales and healthcare services they provided [15–17]. Some efforts have also been made to separate the sale of eyeglasses from optical shops in order to reduce costs, such as facilitating online purchases with provided prescriptions. In addition to the feasibility of employing Standard Patients (SPs) as a methodological approach for assessing the quality of eye examinations and quantifying accuracies in final eyeglasses prescriptions [18–20], optical shops are suitable to detect the impact of separating eyeglasses sales on the eye care quality, thereby providing implications for comprehensive health reform.

We hypothesized that four underlying mechanisms influence the impact of no eyeglasses sale on the quality of eye care, in particularly, the accuracy of the final prescription of the local refractionists provided and the completeness of eye examination. First, supply-induced demand driven by profit motives [21, 22]. Local refractionists may result in prescriptions with higher or lower refraction power, thereby encouraging patients to purchase new eyeglasses. Therefore, refraining from purchasing eyeglasses could mitigate this distorted incentive and enhance the quality of eye care. Second, economic incentives derived from pay-for-performance models [23]. The revenue generated from eyeglasses can act as a strong financial incentive for local refractionists to strive to improve performance. The quality of eye care will decrease while no eyeglasses purchase. Third, if one patient claim no purchase eyeglasses, local refractionists may reallocate their attention to other patients who will purchase eyeglasses, thus resulting in a decline in the quality of eye care [24–26]. Fourthly, not purchasing eyeglasses results in a diminished sense of responsibility from local refractionists towards patients’ ultimate health outcomes [27]. The temporal gap between refraction and eyeglass purchase may inadvertently lead to a perception among local refractionists that there is a reduced necessity to provide high-quality prescriptions in such circumstances.

The aim of this study is to investigate how separating eyeglasses sales from eye examinations affects the accuracy of the final prescription and the potential mechanism analysis. The study will use a randomized controlled trial with incognito standardized patients to evaluate the performance of local refractionists from public and private optical facilities, and compare them with experts from a top eye hospital in China. The study findings will have important implications for healthcare payment policies, leading to better patient care and system efficiency.

## Methods

### Setting

The study was conducted in Shaanxi province, which has a population of 38 million people. Shaanxi is located in the northwestern region of China and has significant wealth disparities. This can be seen from the range of gross domestic product (GDP) per capita among its counties, which ranged from US$5,000 to US$17,531 in 2019, according to the Shaanxi Statistical Yearbook 2020. In terms of GDP per capita, Shaanxi’s value was slightly lower than the overall figure for China, with values of US$9,664 and US$10,279, respectively [28]. There are 10 prefecture-level cities and 107 counties in Shaanxi. Xi’an is the principal capital and the biggest city, accounting for more than one third of the total population and GDP [29].

### Sampling and randomization

We conducted a randomized controlled trial in Shaanxi province to detect the impact of separating eyeglasses sales from eye exams on the quality of eye care. The sample size was determined based on power calculations. Previous literature estimated the inaccuracy of local refractionists to be between 18 to 30% [12, 30]. It was estimated that 89 facilities required in each of the study arms would allow the detection of a 20% difference in the inaccuracy rate of final prescription between intervention and control groups at a 5% level of significance and a power of 80%.

**Fig. 1:**
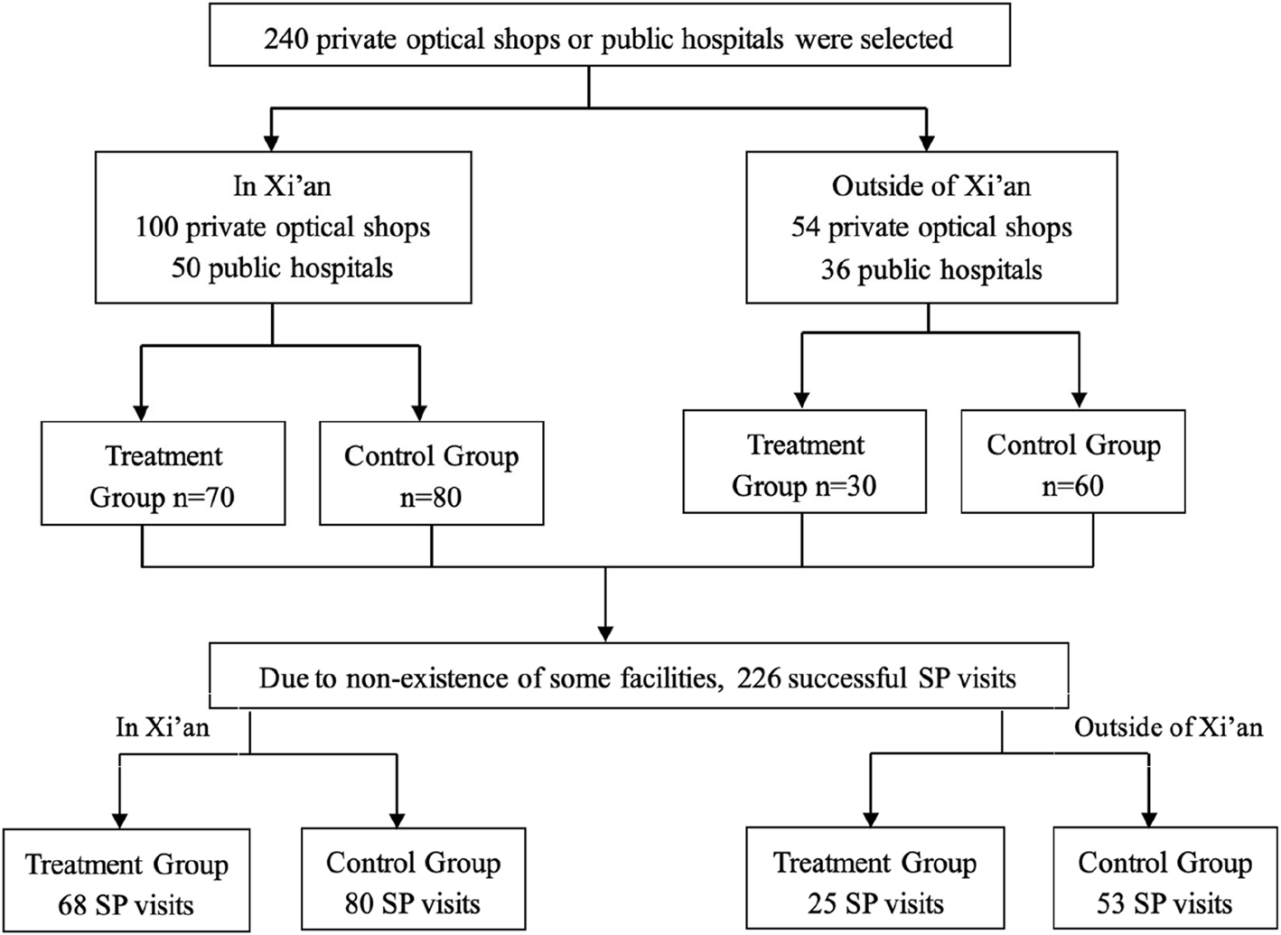
Flow chart of the sample

Our sampling approach targeted both public hospitals equipped with ophthalmology/optometry departments and private optical shops (Fig. 1). We utilized Baidu Maps, a widely-used mapping application in China, to create an extensive list of public and private optical facilities in each city. Given that Xi’an is the provincial capital and the biggest city of Shaanxi and a considerable number of optical chains have their headquarters here, we assigned greater weight to the capital than the remaining regions of the province while sampling. In Xi’an, we randomly selected 100 (accounting for about forty percent) of the eligible private optical shops (*n* = 270) and included 50 public hospitals with ophthalmology departments. This process resulted in a total sample of 150 facilities.

Outside of Xi’an, firstly, we compiled an inclusive list of all 94 counties and districts, and then a random selection of one-fifth (*n* = 18) of these areas was made. On average, each selected county or district had approximately 10 private optical shops and one or two public hospitals with an ophthalmology department. Secondly, within each selected county or district, we randomly selected three private optical shops and two public hospitals with ophthalmology departments, resulting in a total of 90 facilities.

After sampling, we randomly selected 70 facilities in Xi’an (accounting for about 45%) and 30 facilities (one third) outside of Xi’an to form the no eyeglasses sales group, in order to ensure that the treatment group had at least 100 facilities. The remaining facilities, both in Xi’an and outside of Xi’an, were assigned to the control group. During visits, some facilities were found to no longer exist, resulting in a total of 226 successful SP visits, with 148 in Xi’an and 78 outside of Xi’an. Out of these visits, 133 were in the control group and 93 were in the no eyeglasses sales group.

Our study adhered to the principles outlined in the Declaration of Helsinki and obtained ethical approval from the Shaanxi Normal University and Stanford University Institutional Review Board (IRB, Protocol ID 36264). The Institutional Review Board (IRB, Protocol ID 36264) approved the decision not to obtain informed consent from the local refractionists, as it was deemed necessary to preserve the advantage of the incognito approach.

### SP screening and training

To ensure that the SPs were similar to actual patients seen by local opticians in terms of dialect fluency, clothing, and other factors, we recruited 52 graduate students from local counties at Shaanxi Normal University as SPs. These individuals were native speakers of the local dialect and familiar with the cultural norms of the region. One month prior to their visits to local eye care facilities, all SPs underwent a baseline non-cycloplegic eye examination, including visual acuity assessment and automated and subjective refractions performed separately for each eye. Automated refractions (Xinyuan FA-6100, Zhongbei Xinyuan, Taiyuan, China) were performed without the use of cycloplegia, and the best-corrected distance visual acuity and refractive power were automatically output for each eye. The mean of the three automated readings was calculated and used as the starting point for subjective refinement by an ophthalmologist from the Zhongshan Ophthalmologic Center (ZOC), a top-ranking, nationally renowned clinical and research center.

Following the baseline refraction, all SPs underwent three days of intensive training facilitated by researchers from Shaanxi Normal University. The research team, in consultation with ophthalmologists from ZOC, developed the protocols and scripts used during the training. During this period, the SPs memorized the scripts, learned role-playing techniques, and rehearsed behaviors commonly exhibited by real patients. This thorough training process ensured that the SPs closely resembled real patients and could authentically simulate real-life scenarios during their visits to local eye care facilities.

### Facility visits, intervention and data collection

In March 2016, the SPs conducted visits to the selected facilities. To simulate real-world scenarios where patients visit optical shops due to discrepancies between their ideal prescription (referring to the most suitable prescription recommended by the expert ophthalmologists at Zhongshan Ophthalmologic Center in our context) and their current eyeglasses, all SPs were given a pair of eyeglasses with a power that was 0.50 D lower than their actual prescription (specifically, less minus or myopic power). The SPs presented these eyeglasses to the sampled optical providers for assessment.

Each visit was randomly assigned to either the control group or the no eyeglasses sales group. In the control group, the SPs underwent examinations as offered and did not request specific providers. They were instructed to answer all questions during the assessment of visual acuity, automated and subjective refraction, according to their actual ability. If asked, the SPs indicated that they had been experiencing blurry vision for 1–2 weeks, without any accompanying symptoms of headache or other issues. Compared with the control group, the only difference in the no eyeglasses sales group was that the SPs explicitly informed the refractionists that they were not planning to purchase eyeglasses but rather sought an eye examination to assess their visual acuity in advance. Moreover, in advance, SPs in both groups would also request a final formal written prescription for their vision. Eye examinations without eyeglasses purchase will be paid alone if required.

The study aimed to avoid the use of cycloplegia, as it is not permitted at private optical shops in China and is not commonly used on adults at public hospitals. The introduction of cycloplegia could have influenced our results as an uncontrolled variable. Consequently, the SPs were instructed to refuse cycloplegia during their visits if offered (though all offers to use cycloplegia were recorded for documentation purposes).

Upon completion of the visits to the local eye care facilities, the SPs were required to fill out debriefing questionnaires reporting the specific tests they underwent, the prescriptions provided, the examination fees, the total duration of the tests (including assessment of visual acuity, automated and subjective refraction in both eyes, and measurement of the power of existing spectacles), as well as the characteristics of the local refractionists, facilities, and SPs (age, gender, spherical power). To ensure accuracy, prescription and cost information was cross-checked with written documentation received during the visit.

Following the SP visits, a random selection was made of half of the facilities (*n* = 108) for more comprehensive surveys. These surveys included detailed information regarding staff count and facility size, while also evaluating the daily workload of refractionists in terms of the number of refraction cases handled by each refractionist per day.

### Quality measures

We assessed optical providers based on two key quality dimensions observed during their interactions with the SPs: refraction accuracy as the main outcome and process quality as the secondary outcome. The following formula was used to calculate the vector difference in diopters [19, 20], conventionally positive, between the final prescriptions of the local refractionist and the ZOC expert optometrist for each eye of each SP:

$$Vector\ Dioptric\ Distance \left(VDD\right)=\surd 2\times \surd [{\left({SE}_{1}-{SE}_{2}\right)}^{2}+{\left({J0}_{1}-{J0}_{2}\right)}^{2}+{\left({J45}_{1}-{J45}_{2}\right)}^{2}]$$

where SE = spherical equivalent refractive error (sphere + cylinder/2), J0 = - (cylinder power/2) $\times$ cos(2 $\times$ axis), J45 = - (cylinder power/2)$\times$ sin(2 $\times$ axis).

The vector dioptric distance (VDD) is commonly used to measure refraction error [19, 30–32].

The difference of ≥ 1.0 D (absolute value) in either eye between the power prescribed by the local refractionist and the ZOC optometrist was taken as the cutoff for an inaccurate result.

To evaluate process quality, we compared the tests performed by the providers against a clinical checklist derived from the preferred practice patterns (PPP) established by the American Academy of Ophthalmology (AAO), which is officially adopted in China [33]. This checklist included essential optometric procedures such as automatic and subjective refraction while measuring the power of existing spectacles was considered non-essential. In addition to recording the completeness of the eye exam, we also measured the duration of the examination to further evaluate process quality.

### Statistical methods

Descriptive analysis was presented as number (%) for categorical variables and mean (SD) for continuous variables. All univariate comparisons were made using the t-test, Pearson χ2 test or the Fisher exact test. The impact of no eyeglasses sales on prescription accuracy was estimated using multiple logistic regression, while odds ratios (ORs) and corresponding 95% confidence intervals (CIs) were reported as a measurement of the risk. A random intercept model was used to adjust for correlation between eyes of the same SP. Characteristics of SPs, facilities, and local refractionists were included in the regression as potential determinants of inaccurate prescriptions to improve power. Multivariable linear regressions were also performed to estimate the impact of treatment on the duration of eye exams, with the coefficient and corresponding 95% confidence intervals presented. All analyses were performed using Stata 15.0 (StataCorp LP, College Station, TX). All statistical tests were two sided, and *P* < 0.05 was considered statistically significant.

## Results

The SPs underwent a total of 226 eye exams, one each in 73 (32.3%) optical centers from public hospitals and 153 (67.7%) private optical shops (Table 1). Out of the local refractionists, 71 (31.4%) are male. Among the refractionists, 72 (36.3%) were below 30 years old, 55 (24.3%) were 40 years or older, and 89 (39.4%) were aged 30–39 years.

**Table 1:** Characteristics of local refractionists

Variables	Full sample (*n* = 226)	Control Group (*n* = 133)	No sale Group (*n* = 93)
***Characteristics of local refractionists***			
Male, n (%)	71 (31.4)	38 (28.6)	33 (35.5)
Age (years), n (%)			
< 30	82 (36.3)	48 (36.1)	34 (36.6)
30–39	89 (39.4)	52 (39.1)	37 (39.8)
≥ 40	55 (24.3)	33 (24.8)	22 (23.6)
***Facility characteristics***			
Public (vs private), n (%)	73 (32.3)	40 (30.1)	33 (35.5)
Located in Xi’an (provincial capital), n (%)	148 (65.5)	80 (60.2)	68 (73.1)^a^
***SP characteristics***			
Age (years), mean (SD)	24.8 (1.82)	24.7 (1.97)	24.8 (1.59)
Male, n (%)	66 (29.2)	40 (30.1)	26 (28.0)
Spherical equivalent refractive power in the better- seeing eye, n (%)			
> − 3.0 D	111 (49.1)	65 (48.9)	46 (49.4)
≤ − 3.0 D and > − 6.0 D	74 (32.7)	45 (33.8)	29 (31.2)
≤ − 6.0 D	41 (18.1)	23 (17.3)	18 (19.4)
***Accuracy of Final Prescription***			
VDD, mean (SD)	0.802 (0.759)	0.760 (0.725)	0.862 (0.804)
VDD ≥ 1.0 D, n (%)	68 (30.1)	34 (25.6)	34 (36.6)

^a^means the difference between the control and no sale group significantly at a 5% level

In the no sale group, 68 (73.1%) of providers are located in the provincial capital of Xi’an, which is significantly higher than the control group (60.2%). Among the 11 variables tested for characteristics of local refractionists, facilities, and SPs, this is the only one with a significant difference between the two groups. The difference between the final prescription power of local versus expert refractionists was 0.862 D in the no sale group and 0.760 D in the control group (*P* = 0.320). The final prescription inaccuracy rate (differed by an absolute value of ≥ 1.0 D) was 25.6% in the control group and 36.6% in the no sale group (*P* = 0.077).

**Fig. 2:**
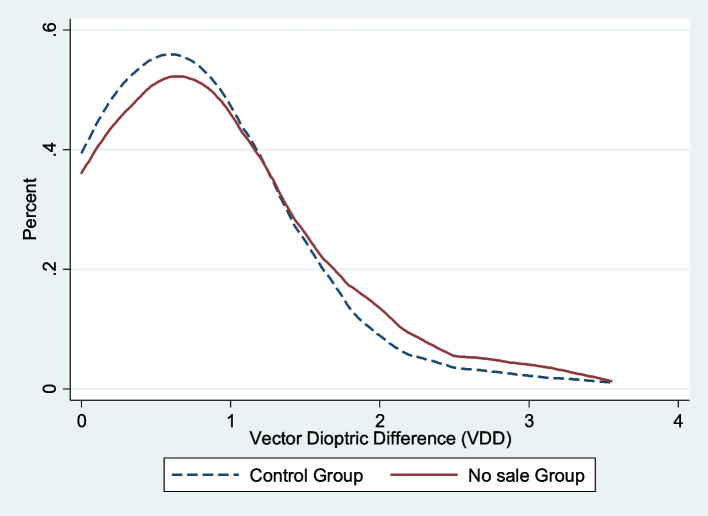
Distribution of VDD between the final prescription power from local refractionists and the expert optometrist in control and no sale group

Figure 2 shows the distribution of the vector difference in diopters (VDD) for the final prescription power given by local refractionists and the expert optometrist in both the control and no-sale groups. The VDD distribution of the no-sale group is shifted more towards the right, indicating a larger value and less accuracy compared to the control group.

The regression analysis results revealed that the likelihood of providing inaccurate final prescriptions was significantly higher in the no-sale group compared to the control group (Table 2; OR = 1.607, *P* = 0.037). This was particularly evident in private optical shops (OR = 2.433, *P* = 0.002), but there was no significant difference in the probability of inaccuracy of the final prescription between the no-sale group and the control group in public hospitals (OR = 0.756, *P* = 0.514).

**Table 2:** Effect of no eyeglasses sales treatment on inaccuracy of final prescription

Final Prescription (VDD ≥ 1.0 D)	Full Sample (*n* = 226)		Private Optical Shops (*n* = 153)		Public Hospitals (*n* = 73)	
	OR (95% CI)	*P* Value	OR (95% CI)	*P* Value	OR (95% CI)	*P* Value
No sale Treatment (control group as reference)	1.607 (1.030 to 2.508)	0.037^*^	2.443 (1.386 to 4.309)	0.002^*^^*^	0.756 (0.327 to 1.751)	0.514
***Characteristics of local refractionists***						
Male	1.45 (0.901 to 2.334)	0.126	1.14 (0.634 to 2.048)	0.661	1.569 (0.619 to 3.979)	0.343
Age (age < 30 as reference)						
30–39	1.108 (0.670 to 1.833)	0.689	0.736 (0.392 to 1.383)	0.341	2.633 (0.976 to 7.101)	0.056
≥ 40	0.623 (0.334 to 1.162)	0.137	0.716 (0.339 to 1.513)	0.381	0.507 (0.127 to 2.017)	0.335
***Facility characteristics***						
Public (vs private)	1.439 (0.902 to 2.294)	0.127				
Located in Xi’an (provincial capital)	1.294 (0.790 to 2.118)	0.306	0.825 (0.454 to 1.498)	0.527	3.379 (1.199 to 9.521)	0.021^*^
***SP characteristics***						
Age (years)	0.898 (0.785 to 1.027)	0.115	0.874 (0.747 to 1.022)	0.092	1.06 (0.779 to 1.442)	0.709
Male	1.399 (0.807 to 2.425)	0.231	1.371 (0.708 to 2.653)	0.350	1.27 (0.408 to 3.948)	0.680
Spherical power, > − 3.0 D as reference						
≤ − 3.0 D and > − 6.0 D	1.348 (0.785 to 2.313)	0.279	1.205 (0.625 to 2.323)	0.579	1.844 (0.632 to 5.379)	0.263
≤ − 6.0 D	4.874 (2.699 to 8.799)	< 0.001^***^	4.074 (1.969 to 8.428)	< 0.001^***^	8.975 (2.858 to 28.188)	< 0.001^***^

^*^P < 0.05

^**^P < 0.01

^***^P < 0.001

Among items in the Preferred Practice Pattern of the American Academy of Ophthalmology (AAO PPP), there was no significant difference between the intervention group and the control group in terms of automatic refraction (Table 3; OR = 0.545, *P* = 0.192). However, refractionists in the no-sale group were significantly less likely to perform subjective refraction compared to the control group (OR = 0.488, *P* = 0.032). Refractionists in the no sale group were also less likely to analyze the spherical power of SP’s current eyeglasses than those in the control group, which is considered to be a non-essential procedure (OR = 0.424, *P* = 0.025). Moreover, the mean duration of eye exams (including assessment of visual acuity, automated and subjective refraction in both eyes) in the no sale group was 3.917 min shorter (*P* = 0.008). Considering that the average duration in the control group was 15.049 min (± 11.645), this effect size corresponds to a substantial reduction of 26.0% compared to the control group.

**Table 3:** Effect of no eyeglasses sales on process completed

Process completed	Essential: Performed Automated refraction		Essential: Performed Subjective refraction		Non-essential: Measured power of existing glasses		Total examination time (min)		
	OR (95%CI)	*P* Value	OR (95%CI)	*P* Value	OR (95%CI)	*P* Value	Beta (95%CI)		*P* Value
No sale Treatment (control group as reference)	0.545 (0.219 to 1.355)	0.192	0.488^*^ (0.253 to 0.940)	0.032^*^	0.424^*^ (0.201 to 0.897)	0.025^*^	-3.917 (-6.798 to -1.036)	0.008^**^	
***Characteristics of local refractionists***									
Male	0.418 (0.153 to 1.139)	0.088	0.766 (0.383 to 1.535)	0.453	0.771 (0.354 to 1.680)	0.513	-1.688 (-4.778 to 1.402)	0.283	
Age (age < 30 as reference)									
30–39	2.106 (0.706 to 6.284)	0.182	1.367 (0.648 to 2.885)	0.412	0.878 (0.379 to 2.033)	0.761	1.373 (-1.914 to 4.660)	0.411	
≥ 40	2.194 (0.612 to 7.873)	0.228	1.072 (0.457 to 2.515)	0.873	0.629 (0.237 to 1.669)	0.352	1.332 (-2.436 to 5.100)	0.487	
***Facility characteristics***									
Public (vs private)	0.175 (0.067 to 0.456)	< 0.001^***^	1.970 (0.961 to 4.037)	0.064	0.057 (0.026 to 0.128)	< 0.001^***^	1.718 (-1.295 to 4.732)	0.262	
Located in Xi’an (provincial capital)	0.552 (0.182 to 1.675)	0.294	2.017 (0.999 to 4.071)	0.050*	1.226 (0.558 to 2.691)	0.612	-0.675 (-3.763 to 2.412)	0.667	
***SP characteristics***									
Age (years)	0.796 (0.568 to 1.115)	0.185	1.036 (0.849 to 1.264)	0.726	1.091 (0.882 to 1.349)	0.423	0.859 (0.013 to 1.705)	0.047	
Male	1.978 (0.604 to 6.472)	0.260	1.585 (0.712 to 3.526)	0.259	0.510 (0.209 to 1.244)	0.139	-0.156 (-3.650 to 3.338)		0.930
Spherical power, > − 3.0 D as reference									
≤ − 3.0 D and > − 6.0 D	3.034 (0.945 to 9.744)	0.062	5.508 (2.317 to 13.095)	< 0.001^***^	0.661 (0.271 to 1.613)	0.364	3.493 (0.131 to 6.855)		0.042^*^
≤ − 6.0 D	11.277 (1.250 to 101.773)	0.031^*^	2.828^*^ (1.129 to 7.082)	0.026^*^	0.904 (0.327 to 2.496)	0.845	3.891 (-0.088 to 7.870)		0.055

^*^P < 0.05

^**^P < 0.01

^***^P < 0.001

The analysis was further stratified based on the workload of local refractionists, determined by whether the number of refractions they conducted daily exceeded the median value (Table 4). The impact of no eyeglasses sale on the inaccuracy of final prescription is slightly higher in subgroups with less busy refractionists (Table 4; OR = 1.585, *P* = 0.408) compared to those with busier refractionists (OR = 1.363, *P* = 0.602). Additionally, the effect size of no eyeglasses sale on the likelihood of performing subjective refraction and total duration of eye examination is also slightly higher in subgroups with less busy refractionists (OR = 0.182, *P* = 0.228; Beta = -4.765, *P* = 0.204) relative to those with busier refractionists (OR = 0.224, *P* = 0.052; Beta = -1.891, *P* = 0.585).

**Table 4:** Effect of no eyeglasses sales stratified by whether the local refractionist busy or not

	Prescription Inaccuracy Rate				Subjective Refraction				Total Examination Time (min)			
	Local Refractionist not Busy		Local Refractionist Busy		Local Refractionist not Busy		Local Refractionist Busy		Local Refractionist not Busy		Local Refractionist Busy	
	OR (95%CI)	*P* Value	OR (95%CI)	*P* Value	OR (95%CI)	*P* Value	OR (95%CI)	*P* Value	OR (95%CI)	*P* Value	OR (95%CI)	*P* Value
No sale Treatment (control group as reference)	1.585 (0.533–4.717)	0.408	1.363 (0.425–4.373)	0.602	0.182 (0.011–2.909)	0.228	0.224 (0.049–1.023)	0.054	-4.765 (-12.246–2.716)	0.204	-1.891 (-8.809–5.027)	0.585
***Characteristics of local refractionists***												
Male	3.788 (1.318–10.888)^*^	0.013	0.765 (0.212–2.759)	0.682	0.225 (0.032–1.566)	0.132	1.034 (0.178–5.989)	0.970	1.993 (-5.080–9.067)	0.570	-1.753 (-9.483–5.978)	0.651
Age (age < 30 as reference)												
30–39	1.027 (0.355–2.972)	0.961	1.588 (0.376–6.700)	0.529	0.542 (0.057–5.147)	0.593	0.550 (0.081–3.749)	0.542	3.366 (-3.627–10.360)	0.334	2.054 (-6.489–10.598)	0.631
≥ 40	0.963 (0.235–3.949)	0.958	0.904 (0.169–4.847)	0.907	0.520 (0.012–22.876)	0.735	0.849 (0.096–7.494)	0.883	1.932 (-7.271–11.135)	0.672	-0.136 (-9.268–8.996)	0.976
***Facility characteristics***												
Public (vs private)	3.282(0.901–11.953)	0.071	1.277 (0.360–4.529)	0.705	1.000 (1.000–1.000)	0.999	1.708 (0.348–8.390)	0.510	13.578 (0.390–26.765)^*^	0.044	4.274 (-2.846–11.395)	0.234
Located in Xi’an (provincial capital)	1.972(0.507–7.674)	0.328	0.532 (0.086–3.291)	0.497	10.160 (0.217–475.299)	0.237	3.841 (0.227–53.242)	0.316	0.911 (-7.297–9.118)	0.823	-2.040 (-13.014–8.934)	0.710
***SP characteristics***												
Age (years)	0.862 (0.656–1.132)	0.286	1.046 (0.667–1.641)	0.844	0.830 (0.454–1.518)	0.545	1.035 (0.626–1.712)	0.893	1.235 (-0.593–3.062)	0.178	1.854 (-0.221–3.929)	0.079
Male	3.193 (0.902–11.310)	0.072	1.092 (0.193–6.183)	0.921	1.615 (0.078–33.387)	0.756	0.595 (0.063–5.605)	0.650	-0.176 (-8.361–8.010)	0.965	1.434 (-8.508–11.376)	0.773
Spherical power, > -3.0 D as reference												
≤ − 3.0 D and > − 6.0 D	0.870 (0.258–2.941)	0.823	2.251 (0.574–8.819)	0.244	28.446 (0.527–1534.933)	0.100	5.896 (0.962–36.150)	0.055	3.201 (-4.940–11.342)	0.429	3.854 (-4.108–11.816)	0.336
≤ − 6.0 D	5.082 (1.013–25.480)*	0.048	2.564 (0.462–14.229)	0.281	9.772 (0.108–885.947)	0.322	1.000 (1.000–1.000)	0.999	-1.716 (-12.847–9.414)	0.755	7.233 (-2.995–17.460)	0.162

This analysis is solely based on the facilities that had participated in the facilities survey

^*^P < 0.05

## Discussion

This study evaluated the quality of local eye care providers using standardized patient methods in a randomized controlled trial. The interactions between incognito SPs and local refractionists showed that the quality of eye care provided in both public and private optical facilities is quite low. The local prescription inaccuracy rate was found to be as high as 25.6% in control group, with a difference of ≥ 1 D in prescribed power as the cutoff for an inaccurate result. Considering our study has refuted the plausibility of induced-demand (to be discussed in detail below), based on previous research findings, we postulate that the limited training provided to optical providers and regulatory restrictions prohibiting non-physicians from administering medical cycloplegic agents may contribute to the observed high rate of prescription inaccuracy [12, 30]. Despite the increased accessibility of optical services due to economic and societal development, there is still a need for improvement in the quality of eye care.

Our study suggests that separating eyeglasses sales from optical care could lead to worse quality of eye care in terms of the inaccuracy rates of the final prescription. Eyeglasses sales generate the most profit for optical shops and hospitals. While no eyeglasses sale reform may be initially intended to reduce consumers’ expenditure on eyeglasses, our findings indicate that it could lead to unintended consequences, with the inaccuracy rate of final prescriptions increasing by 1.6 times. This effect was more profound in private healthcare settings, where the inaccuracy rate increased by up to 2.4 times due to the lack of eyeglasses sales. This result should make sense because private healthcare providers are usually considered as relying more on revenue from drug sales [5]. These findings may also offer insights into the reform in broader healthcare domains. In China, hospitals heavily depend on revenue generated from drug sales and the provision of healthcare services [34]. To reduce the heavy reliance of public hospitals on drug sales and to contain the escalating medical expenditures, comprehensive health reform focusing on separating drug sales from hospital revenue has been introduced in China [5, 35].

We further investigated the potential mechanism underlying these findings. Firstly, our results did not support the induced-demand hypothesis as the observed decline in eye care quality due to a lack of eyeglasses sales contradicted expectations associated with this mechanism.

Secondly, our findings further revealed that the quality of the process of eye examinations also decreased without economic incentives from eyeglasses sales, thereby aligning more closely with the pay-for-performance mechanism. Local refractionists may exhibit less motivation to improve their performance without additional rewards. We conducted an examination of the completeness of assessments called for in standard international protocols which have been officially adopted in China. Subjective refinement of an automated value by an experienced practitioner is considered a gold standard for refraction, and should tend to reduce errors due to instrument accommodation if done correctly [33]. However, we found that subjective refraction is only performed about half as often in the no eyeglasses sales group compared to the control group. Moreover, the local refractionists in the no eyeglasses sales group seem less motivated to measure the power of SPs’ existing eyeglasses, despite it being a non-essential process. The shorter duration of eye exams provided more comprehensive evidence that local refractionists in the no eyeglasses sales group may not be giving their best effort during eye exams as they usually do.

Thirdly, our findings do not support the hypothesis that the time allocated to attending to other regular patients is responsible for the decline in quality of eye care, despite its potential theoretical explanatory power. This mechanism only holds true when local refractionists are overwhelmed and have to serve multiple patients simultaneously. Nevertheless, regardless of whether the local refractionists was busy or not, we observed no significant variation in the performance of local refractionists, including both the accuracy of their final prescriptions and the completeness of eye examinations.

Finally, we posit that the mechanism of diminishing responsibility would exert minimal influence on our findings. In cases where a prescription lacks relevance to a patient’s subsequent treatment, healthcare providers may perceive themselves as not being accountable for patients’ ultimate health outcomes and consequently reduce their efforts in providing accurate prescriptions. Although our experimental design was not specifically tailored to directly examine this mechanism, two supporting facts can be highlighted. Firstly, the SPs were instructed to request formal written final prescriptions in order to prevent local refractionists from perceiving these patients as undeserving of careful treatment. Secondly, the observation that the impact was more pronounced for private optical shops aligns better with the economic incentive mechanism mentioned above rather than with this mechanism, since the latter cannot account for such differences between private and public optical shops.

In summary, our findings provide stronger support for the pay-for-performance mechanism and highlight the importance of economic incentives in promoting high quality of healthcare.

The findings of this paper have several significant implications for policymaking in optical care as well as the whole healthcare sector. Firstly, it is crucial to improve not only the accessibility but also the quality of optical care. Furthermore, based on the underlying mechanism, this paper presents a more extensive perspective on healthcare reform, particularly in separating drug sales from hospital revenue. While high profits in the healthcare sector can lead to undesirable outcomes such as overtreatments, removing all economic incentives may negatively impact doctors’ motivation and, thus, the quality of care. Human actions are driven by underlying motivations, and the revenue generated through drug sales serves as a powerful economic incentive for healthcare providers to strive for enhanced performance over time. Any decline in the quality of care provided would ultimately lead to reduced financial gains. Therefore, policy makers should carefully consider the role of economic incentives in healthcare reform [5, 11]. For example, certain adjustments could be contemplated to incorporate into the current reform in order to maintain the proper economic incentives for doctors [5, 16]. This may involve appropriately augmenting the registration fee for doctors’ diagnostic services in the absence of further drug sales, or providing supplementary subsidies based on their diagnostic performance if hospitals effectively adhere to the zero markup policy on medical consumables.

The strength of this paper lies in its measurement and identification methods. Previous literature on the reform regarding the separation of drug sales from hospital revenue has primarily focused on its impact on patients’ total expenditure [5, 7–9]. Although the impact on the quality of healthcare is also important, the lack of studies in this area is due to difficulties in measuring healthcare quality and identifying causal effects. To fill this gap, our study introduces SPs, which are referred to as the best method for measuring real quality of medical care performance [11, 36], and RCTs, which are referred to as the gold standard for evaluating policy impact [37]. Thus, to our knowledge, it is the first to investigate the impact of the separation of drug sales from hospital revenue on healthcare quality, making it a valuable contribution to the field.

We need to acknowledge the limitations of this paper. Firstly, we faced difficulty in collecting sufficient basic information about local optical providers, which made it challenging to clarify how optometry service providers with different characteristics respond to the separation of medicine. However, we believe that the robustness of the study findings remains unaffected by this limitation, as there were no statistically significant differences observed in the distribution of all known characteristics and potential confounders between the groups (except for one) (Table 1). Therefore, it is unlikely that any unknown factors were unevenly distributed between both groups. Secondly, we only focused on eye care, which may have different characteristics from other healthcare sectors, thus affecting the generalizability of the results to the entire healthcare industry. Thirdly, the main samples were carried out in only one western province, which also limits the expansibility of the results to other areas.

## Data Availability

The datasets supporting the conclusions of this study are available from the corresponding author upon reasonable request.
